# Can clinical factors at presentation be used to predict outcome of treatment with methotrexate in patients with early inflammatory polyarthritis?

**DOI:** 10.1136/ard.2008.088237

**Published:** 2008-02-21

**Authors:** S L Hider, A J Silman, W Thomson, M Lunt, D Bunn, D P M Symmons

**Affiliations:** arc Epidemiology Unit, University of Manchester, UK

## Abstract

**Purpose:**

Methotrexate (MTX) is the first choice conventional disease-modifying antirheumatic drug (DMARD) for rheumatoid arthritis. It is not universally effective, however; although to date it is not possible to predict with any accuracy which patients will respond to treatment. The aim of this analysis was to examine whether clinical and genetic variables could be used to predict response to MTX.

**Methods:**

Patients recruited to the Norfolk Arthritis Register (NOAR), a primary care based inception cohort of patients with inflammatory polyarthritis, were eligible for this analysis if they were commenced on MTX as their first DMARD within 3 months of their baseline visit and had at least 2 years of follow-up data. Outcome on MTX was defined as: (1) stopped for adverse events; (2) stopped for inefficacy or second DMARD added; (3) stopped for other reasons; or (4) remained on MTX monotherapy. Multiple logistic regression was used to establish which variables (including demographics, disease activity and Health Assessment Questionnaire score) predicted stopping monotherapy for inefficacy or adverse event (with those remaining on treatment taken as the referent category). The area under the Receiver Operating Characteristic curves (AUC ROC), were used to determine how accurate the model was at predicting outcome.

**Results:**

309 patients were included in this analysis. At 1 year (2 years), 34 (46) patients had stopped for adverse events and 25 (49) had either stopped monotherapy for inefficacy or had a second DMARD added. 231 (188) patients remained on MTX monotherapy. The strongest predictor of inefficacy at both time points was shared epitope positivity: odds ratios (OR) 5.8 (95% confidence intervals (CI) 1.3 to 25.6) at 1 year, OR 3.0 (95% CI 1.3 to 7.3) at 2 years. High Health Assessment Questionnaire score (OR 1.84 95% CI 1.12 to 3.01) and female gender (OR 2.2, 95% CI 0.92 to 5.28) were associated with adverse events on MTX at 1 year. However, even the most optimal combinations of the factors analysed were only weakly predictive of treatment outcome: AUC ROC for adverse events 0.68 (95% CI 0.58 to 0.78) and for inefficacy AUC ROC 0.71 (95% CI 0.6 to 0.81).

**Conclusions:**

Within this cohort, routine clinical and laboratory factors were poor at predicting outcome of treatment with MTX. Given the major therapeutic advantage to be derived from accurate prediction of treatment outcome, further studies will need to investigate novel biological and other markers.

Methotrexate (MTX) is the key conventional disease-modifying antirheumatic drug (DMARD) treatment for rheumatoid arthritis (RA) as it is cheap, effective and can be prescribed as monotherapy or in combination with other agents, including biologicals. Studies suggest it is now the first DMARD of choice for RA worldwide.^1^ Recent studies have done much to confirm its clinical efficacy with ACR50 responses between 46% and 65%.^2^ ^3^ In addition, patients remain on MTX significantly longer than other conventional DMARDs.^4^ ^5^

MTX is, however, not universally effective, and a significant proportion of patients stop treatment because of inefficacy or adverse events. There are, however, few data as to the possibility of being able to predict patients’ response to treatment. As patients usually have to fail treatment with MTX prior to the prescription of biologicals it would be particularly useful to be able to predict treatment outcome, in that patients unlikely to respond to treatment could be fast-tracked to drugs from which they would be more likely to derive benefit.

Although outcome studies have identified RA-related factors (eg, high erythrocyte sedimentation rate/C-reactive protein (CRP), rheumatoid factor, shared epitope (SE) positivity and extra-articular disease) to be associated with an adverse prognosis in RA in terms of radiographic damage or disability,^6^ to date few studies have examined the effect of such factors on treatment outcome. Anderson *et al*^7^ identified disease duration as the strongest predictor of treatment response in a meta-analysis of 1435 patients, with patients with longer disease duration at the start of treatment being less likely to respond to MTX. Other factors suggested to be associated with poorer response include previous DMARD use^7^ ^8^ and poor functional status.^7^ Clearly these factors may be, in part, confounded by disease duration, in that patients with a longer duration of disease will usually have been exposed to more drugs and have had longer to accrue physical disability. Similarly, failure with other DMARDs might indicate drug-resistant disease, or disease less likely to respond to treatment.

Studies examining disease activity as a predictor of MTX response are inconsistent, with some studies suggesting that patients with low disease activity are less likely to respond to treatment.^7^ ^8^ However, in a study published by Hoekstra *et al*,^9^ patients with low disease activity at baseline were *more* likely to respond to treatment with MTX. In part, these differences may reflect the different definitions of disease activity used, in that some studies used inflammatory markers as a marker of disease activity,^8^ whereas others used composite measures such as the disease activity score.^9^

To minimise the effect of increasing disease duration and determine whether clinical variables influence MTX response it is necessary to study patients early in their disease course. The Norfolk Arthritis Register (NOAR) provides an ideal population for further study as patients are recruited at the time of diagnosis, when data are recorded on disease activity and severity and patients are followed annually. The aim of this analysis was to examine whether clinical factors could be used to predict outcome of treatment with MTX used as the first DMARD in patients with early inflammatory polyarthritis (IP).

## PATIENTS AND METHODS

### Subjects

Subjects were recruited from NOAR, which is a primary care based inception cohort of patients with early IP. Details of the register have been published elsewhere.^10^ Briefly, NOAR aims to recruit all new cases of IP from a large defined geographical area of approximately 400 000 adults (age ⩾16 years). Any individual presenting to their primary care physician with swelling of two or more joints lasting at least 4 weeks should be notified to NOAR. A parallel notification system operates from hospitals within the catchment area. All patients who are referred to secondary care are treated by one of four rheumatologists according to their routine clinical practices. For the purposes of this analysis all patients recruited to NOAR between 1 January 1995 and 31 December 2003 who were commenced on MTX as their first DMARD within 3 months of their baseline visit were eligible for inclusion. Clinical guidelines exist for the management of patients on MTX, patients are started on a standard dose MTX (7.5–10 mg/week) and monitored in accordance with UK national guidelines, with dose escalation (up to 25 mg/week) at the discretion of the treating rheumatologist. The study was approved by the local research ethics committee and all patients provided written consent. Patients were included in this analysis if they were treated with MTX as their first DMARD within 3 months of their baseline assessment and had 2 years of follow-up data.

### Baseline assessment

One of a team of trained research nurses performed a structured interview and clinical examination at the baseline and follow-up assessments. Clinical and demographic data recorded included age at symptom onset, gender, symptom duration, tender joint count (TJC, 53 joints) and swollen joint count (SJC, 51 joints). All patients also completed a Health Assessment Questionnaire (HAQ), modified for use in British patients.^11^ At baseline, blood samples were taken for rheumatoid factor (RF) and C-reactive protein (CRP) measurement and DNA was extracted for SE status. RF was measured using a latex agglutination technique and a titre of ⩾1:40 was considered positive. SE status was examined using the Dynal RELI SSO HLA-DRB1 Typing kit (Dynal, Bromborough, UK). The disease activity score (using 28 joint counts) (DAS28) was calculated for each patient using the 28 SJC, 28 TJC and CRP using the formula (DAS28 = (0.56×SQRT(TJC28)+0.28×SQRT(SJC28)+0.36×ln(-CRP+1))×1.10+1.15) (from Flendrie and Fransen http://www.umcn.nl/userfiles/other/dasculators.xls/ accessed 23 September 2008).

#### Follow-up

Patients were then followed up annually using a nurse-administered questionnaire and HAQ. This records medication details, including start and stop date of DMARDs and reason for stopping treatment. For this study the patient reported reason for stopping treatment was validated by medical record and laboratory data review. Outcome on MTX was then classified as either: (1) stopped for an adverse event; (2) stopped for inefficacy (physician statement) or had second DMARD added; (3) stopped for other reasons; or (4) remained on treatment.

### Statistical analysis

The baseline characteristics were compared between those who stopped MTX treatment at 1 and at 2 years with those who continued treatment at each time-point. Comparison of continuous data was carried out using the Mann–Whitney U test. For categorical data the χ^2^ test was employed. Logistic regression models were used to assess the relationship between predictor variables and the event of interest (either inefficacy or adverse event) compared with remaining on MTX monotherapy (which was taken as the referent category). An analysis that examined predicting MTX failure (for either inefficacy or adverse events) compared with remaining on MTX monotherapy was also performed. The areas under the resulting Receiver Operating Characteristic curve (AUC ROC, were used to determine how accurate the models were at predicting outcome. All analyses were performed using STATA 8.0 (STATA Corporation, College Station, Texas, USA, 1993).

## RESULTS

**Figure 1:**
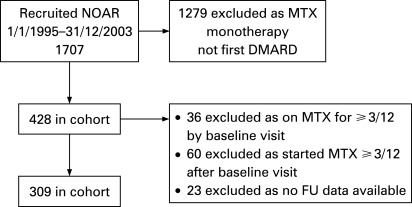
How the patient cohort was derived. DMARD, disease-modifying antirheumatic drug; FU, follow-up; MTX, methotrexate; NOAR, Norfolk Arthritis Register.

**Figure 2:**
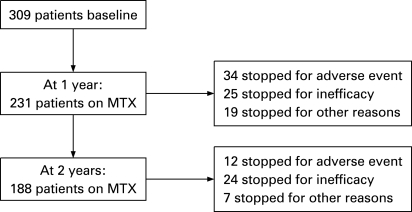
Outcome of methotrexate (MTX) monotherapy treatment at 1 and 2 years.

There were 309 patients who were eligible to be included in this analysis. During the period of recruitment, 1707 patients were ascertained by NOAR and the derivation of the 309 patients included in this analysis is shown in fig 1. These were a typical population with IP in that 66% were female, and median age at symptom onset was 59.3 (interquartile range 48.3–69 years). As expected there was a range of disease activity at baseline, median DAS28 3.9 (interquartile range 3.0–4.8). At baseline 51% of the cohort fulfilled American Rheumatism Association criteria for RA,^12^ which increased to 70% at 1 year. Figure 2 shows the outcome of MTX monotherapy at 1 and 2 years, illustrating that treatment survival was good with 75% and 61% of patients remaining on monotherapy at 1 and 2 years respectively. Perhaps not surprisingly, more patients stopped monotherapy because of adverse events in the first year and because of inefficacy in the second. There was a small group of patients who stopped for other reasons (including planning pregnancy and patient choice). This latter group was excluded from further analyses.

### Predicting inefficacy

Cumulatively, 25 patients stopped MTX monotherapy for inefficacy in the first year, and 49 patients had stopped monotherapy at 2 years. Of these 49 patients, 35 had a second DMARD added. Baseline clinical and demographic variables of those stopping MTX monotherapy for inefficacy were similar to those remaining on treatment (table 1).

**Table 1:** Predicting inefficacy at 1 and 2 years

Predictor	1 year			2 years		
	Inefficacy	Still on	p Value	Inefficacy	Still on	p Value
Number of patients N (%)	25 (8)	231 (75)		49 (16)	188 (61)	
Female gender N (%)	18 (72)	147 (64)	0.8	33 (67)	117 (62)	0.5
Age diagnosis (years)	60.6 (53.3–73.1)	59.3 (48.3–69)	0.34	60.6 (52.5–68.6)	58.7 (46.9–69.5)	0.43
Age MTX onset (years)	61.2 (53.9–73.2)	60 (50.8–69.8)	0.38	61.2 (53.1–68.7)	59.4 (49.1–70.5)	0.55
Symptom duration—prior MTX (months)	6.01 (3.35–8.01)	6.27 (3.75–12.68)	0.38	6.01 (3.35–7.95)	6.09 (3.56–14.59)	0.11
RF positive (yes/no)	14 (56)	94 (41)	0.12	25 (51)	87 (46)	0.25
ARA criteria (yes/no)	11 (44)	121 (52)	0.43	25 (51)	101 (54)	0.74
SJC	7 (2–10)	4 (1–11)	0.8	5 (2–12)	5 (1–11)	0.72
TJC	4 (1–12)	5 (1–15)	0.94	4 (0–17)	5 (1–13)	0.77
28 SJC	4 (1–9)	4 (1–9)	0.84	4 (1–10)	4 (1–9)	0.64
28 TJC	3 (0–7)	3 (0–10)	0.28	3 (0–7)	4 (0–10)	0.26
Baseline CRP*	20 (6–36)	13 (5–26)	0.33	20 (6–38)	13 (5–26)	0.26
Baseline DAS28*	3.7 (2.8–4.7)	3.9 (3.0–4.8)	0.59	3.7 (2.8–4.8)	4.0 (3.0–4.8)	0.67
Baseline HAQ	1 (0.375–2)	1 (0.5–1.625)	0.57	1.125 (0.5–1.875)	1 (0.5–1.625)	0.34
SE positive (yes/no)† N (%)	21 (91)	126 (64)	0.009	37 (84)	99 (63)	0.01
Steroids (yes/no)	6 (24)	39 (17)	0.38	19 (38)	18 (10)	0.001

ARA, American Rheumatism Association; CRP, C-reactive protein; DAS, disease activity score (using 28 joint counts); HAQ, Health Assessment Questionnaire; MTX, methotrexate; RF, rheumatoid factor; SE, shared epitope; SJC, swollen joint count; TJC, tender joint count.

This table illustrates clinical variables in those stopping for inefficacy and those remaining on treatment at 1 and 2 years. Results expressed as median (IQR) unless stated.

*293 patients had baseline CRP available and, therefore, could have DAS28 results calculated.

†260 patients had DNA samples and were successfully genotyped for SE.

By contrast, possession of the SE was associated with MTX inefficacy at both 1 year (unadjusted OR 5.83 95% CI 1.33 to 25.6, p = 0.02) and 2 years (unadjusted OR 3.04 95% CI 1.27 to 7.27, p = 0.01). Adjusting for age, gender, symptom duration and steroid use did not attenuate this association (table 2).

**Table 2:** Predicting inefficacy

Predictor	1 year				2 years			
	Unadjusted OR (95% CI)	p Value	Adjusted OR (95% CI)	p Value	Unadjusted OR (95% CI)	p Value	Adjusted OR (95% CI)	p Value
SE positive	5.83 (1.33 to 25.61)	0.02	5.88 (1.32 to 26.25)	0.02	3.04 (1.27 to 7.27)	0.01	3.04 (1.21 to 7.62)	0.02

MTX, methotrexate; SE, shared epitope.

This table illustrates the odds of stopping MTX for inefficacy—OR (95% CI) displayed as unadjusted and adjusted for age, gender, symptom duration and steroid use at baseline.

However, using the ROC curve, SE positivity was only moderate in predicting MTX inefficacy at 12 months, AUC ROC 0.64 (95% CI 0.48 to 0.75). By including all other variables that showed some evidence of possible association (table 2) as well as age, gender, symptom duration and steroid use did not significantly improve the predictive power of the model, AUC ROC 0.7 (95% CI 0.6 to 0.81). The results were similar at 2 years unadjusted AUC ROC 0.6 (95% CI 0.54 to 0.67) and adjusted for the same variables AUC ROC 0.74 (95% CI 0.65 to 0.81).

### Predicting adverse events

Thirty-four patients stopped MTX for adverse events in the first year, this increased to 46 patients by 2 years. Baseline clinical and demographics of the adverse event group compared with those remaining on treatment are shown in table 3.

**Table 3:** Predicting adverse events at 1 and 2 years

Predictor	1 year			2 years		
	Adverse event	Still on	p Value	Adverse event	Still on	p Value
Number of patients	34 (11)	231 (75)		46 (15)	188 (61)	
Female gender n (%)	27 (79)	147 (64)	0.07	35 (76)	117 (62)	0.08
Age diagnosis (years)	58.4 (44.5–64.4)	59.3 (48.3–69)	0.25	59 (45.1–65.6)	58.7 (46.9–69.5)	0.56
Age MTX onset (years)	58.8 (45.6–64.7)	60 (50.8–69.8)	0.29	59 (45.8–68.0)	59.4 (49.1–70.5)	0.6
Symptom duration prior to MTX (months)	8.76 (4–18.95)	6.27 (3.75–12.68)	0.21	6.97 (4.01–16.79)	6.09 (3.56–14.59)	0.33
RF positive (yes/no)	11 (32)	94 (41)	0.31	15 (44)	87 (46)	0.2
ARA criteria (yes/no)	16 (47)	121 (52)	0.56	20 (44)	101 (54)	0.2
SJC	4 (2–10)	4 (1–11)	0.83	4 (2–9)	5 (1–11)	0.43
TJC	9 (2–15)	5 (1–15)	0.06	8 (3–16)	5 (1–13)	0.08
28 SJC	3 (2–8)	4 (1–9)	0.99	3 (1–7)	4 (1–9)	0.4
28 TJC	6 (3–9)	3 (0–10)	0.05	6 (2–11)	4 (0–10)	0.09
Baseline CRP*	8 (2–17)	13 (5–26)	0.04	7 (2–17)	13 (5–26)	0.01
Baseline DAS28*	3.8 (3.3–5.1)	3.9 (3.0–4.8)	0.65	3.6 (3.3–4.9)	4.0 (3.0–4.8)	0.86
Baseline HAQ	1.625 (0.875–2)	1 (0.5–1.625)	0.02	1.25 (0.875–2)	1 (0.5–1.625)	0.1
SE positive (yes/no)†	19 (68)	126 (64)	0.71	25 (63)	99 (63)	0.91
Steroids (yes/no)	5 (15)	39 (17)	0.75	9 (20)	18 (10)	0.06

ARA, American Rheumatism Association; CRP, C-reactive protein; DAS, disease activity score (using 28 joint counts); HAQ, Health Assessment Questionnaire; MTX, methotrexate; RF, rheumatoid factor; SE, shared epitope; SJC, swollen joint count; TJC, tender joint count.

This table illustrates clinical variables in those stopping for adverse events and those remaining on treatment at 1 and 2 years. Results are expressed as median (IQR) unless stated.

*293 patients had baseline CRP available and, therefore, could have DAS28 results calculated.

†260 patients had DNA samples and were successfully genotyped for SE.

At 1 year, patients stopping MTX for adverse events had a higher baseline median HAQ score (1.625 vs 1, p = 0.02), higher TJC (9 vs 5, p = 0.05) but lower CRP (8 vs 13, p = 0.04) than those remaining on treatment. There was also a trend for female patients to be more likely to stop MTX for adverse events (79% vs 64%, p = 0.07), although this did not reach statistical significance. By 2 years, only lower CRP (7 vs 13, p = 0.01) was associated with stopping MTX for adverse events, although a trend towards a higher TJC in those stopping for adverse events remained. No association was observed between SE status and adverse events on MTX. Using logistic regression, higher baseline HAQ score was associated with stopping MTX for adverse events at 1 year (unadjusted OR 1.84 95% CI 1.12 to 3.01, p = 0.02). Adjusting for age, gender, symptom duration and steroid use did not affect this association (table 4).

**Table 4:** Predicting adverse events

Predictor	1 year				2 years			
	Unadjusted OR (95% CI)	p Value	Adjusted OR (95% CI)	p Value	Unadjusted OR (95% CI)	p Value	Adjusted OR (95% CI)	p Value
HAQ score	1.84 (1.12 to 3.01)	0.02	1.87 (1.11 to 3.15)	0.02	1.47 (0.94 to 2.29)	0.09	1.42 (0.89 to 2.26)	0.14

HAQ, Health Assessment Questionnaire.

This table illustrates OR of stopping methotrexate for adverse events—OR displayed as unadjusted and adjusted for age, gender, symptom duration and steroid use at baseline.

Despite this association, however, HAQ was only moderate in predicting adverse events on MTX, AUC ROC unadjusted 0.63 (95% CI 0.52 to 0.73) and 0.68 (95% CI 0.58 to 0.78) when adjusted for age, gender, symptom duration and steroid use. Adding in the other factors significant on univariate analysis (TJC and CRP) did not further improve the predictive power of the model AUC ROC 0.69 (95% CI 0.6 to 0.79). At 2 years, the results were similar, with a trend for higher HAQ to be associated with stopping MTX for adverse events. This no longer reached statistical significance (table 4).

### Predicting methotrexate failure overall

Fifty-nine patients failed MTX monotherapy at 1 year, this increased to 95 patients by 2 years. Baseline clinical and demographics of the failure group compared with those remaining on MTX monotherapy are shown in table 5. At 1 year, patients failing MTX monotherapy had a higher baseline median HAQ score (1.5 vs 1, p = 0.04) and were more likely to be SE positive (78% vs 64%, p = 0.05) than those remaining on monotherapy, although neither of these associations remained statistically significant at 2 years. Again these factors were poor at predicting MTX failure, AUC ROC HAQ unadjusted 0.59 (95% CI 0.5 to 0.68) and 0.63 (95% CI 0.55 to 0.72) when adjusted for age, gender, symptom duration and steroid use (table 6).

**Table 5:** Predicting MTX failure

Predictor	1 year			2 years		
	MTX fail	Still on	p Value	MTX fail	Still on	p Value
Number of patients	59 (25)	231 (75)		95	188 (61)	
Female gender n (%)	45 (76)	147 (64)	0.07	68 (72)	117 (62)	0.1
Age diagnosis (years)	58.9 (45.4–70.5)	59.3 (48.3–69)	0.8	59.3 (52–68.2)	58.7 (46.9–69.5)	0.88
Age MTX onset (years)	58.9 (47.8–71)	60 (50.8–69.8)	0.82	59.9 (52.2–68.8)	59.4 (49.1–70.5)	0.95
Symptom duration prior to MTX (months)	6.24 (3.6–13.18)	6.27 (3.75–12.68)	0.72	6.28 (3.64–13.04)	6.09 (3.56–14.59)	0.66
RF positive (yes/no)	25 (45)	94 (41)	0.84	40 (44)	87 (46)	0.95
ARA criteria (yes/no)	27 (46)	121 (52)	0.36	45 (47)	101 (54)	0.3
SJC	5 (2–10)	4 (1–11)	0.76	5 (2–10)	5 (1–11)	0.79
TJC	8 (2–15)	5 (1–15)	0.19	6 (1–16)	5 (1–13)	0.35
28 SJC	3 (2–8)	4 (1–9)	0.89	3 (1–8)	4 (1–9)	0.83
28 TJC	5 (1–9)	3 (0–10)	0.46	4 (0–9)	4 (0–10)	0.73
Baseline CRP*	10 (2–26)	13 (5–26)	0.38	10 (3–28)	13 (5–26)	0.86
Baseline DAS28*	3.71 (3.1–4.9)	3.9 (3.0–4.8)	0.99	3.7 (3.1–4.9)	4.0 (3.0–4.8)	0.69
Baseline HAQ	1.5 (0.625–2)	1 (0.5–1.625)	0.04	1.25 (0.625–1.875)	1 (0.5–1.625)	0.1
SE positive (yes/no)†	40 (78)	126 (64)	0.05	62 (74)	99 (63)	0.1
Steroids (yes/no)	11 (18.6)	39 (16.9)	0.75	28 (29)	18 (10)	0.001

ARA, American Rheumatism Association; CRP, C-reactive protein; DAS, disease activity score (using 28 joint counts); HAQ, Health Assessment Questionnaire; MTX, methotrexate; RF, rheumatoid factor; SE, shared epitope; SJC, swollen joint count; TJC, tender joint count.

*293 patients had baseline CRP available and, therefore, could have DAS28 results calculated.

†260 patients had DNA samples and were successfully genotyped for SE.

**Table 6:** Predicting methotrexate failure

Predictor	1 year				2 years			
	Unadjusted OR (95% CI)	p Value	Adjusted OR (95% CI)	p Value	Unadjusted OR (95% CI)	p Value	Adjusted OR (95% CI)	p Value
HAQ score	1.55 (1.05 to 2.3)	0.03	1.47 (0.98 to 2.2)	0.06	1.35 (0.97 to 1.9)	0.08	1.34 (0.93 to 1.93)	0.1
SE positive	2.02 (0.98 to 4.18)	0.05	2.5 (1.2 to 5.47)	0.02	1.62 (0.9 to 2.9)	0.1	1.75 (0.94 to 3.27)	0.08

HAQ, Health Assessment Questionnaire; SE, shared epitope.

## DISCUSSION

This cohort provides an insight into the “real world” response to MTX within an unselected inception cohort of patients with IP starting MTX as their first DMARD. Although the total numbers starting MTX as their first DMARD (with clinical data within 3 months of drug start) was relatively small this analysis provides additional evidence that MTX is effective and generally well tolerated in an unselected IP cohort, with 75% of patients remaining on monotherapy at 1 year and 61% at 2 years. Within this cohort, standard clinical and laboratory variables collected at first visit were poorly predictive of treatment outcome. Interestingly, the most strongly associated predictor of MTX inefficacy at both 1 and 2 years was possession of the SE, whereas other variables, such as CRP, DAS28 and HAQ, did not appear to be associated with inefficacy. The majority of patients stopping for adverse events did so within the first 12 months, within this group higher HAQ score, higher TJC and lower CRP were associated with stopping treatment for adverse events. However, for neither outcome were these variables, even when used in combination, sufficiently predictive of outcome to be of clinical use.

Studies suggest that high levels of inflammation (as measured by erythrocyte sedimentation rate, CRP or joint counts) predict a poor prognosis in terms of radiological damage.^13^ Our results suggest that disease activity does not influence treatment response in either direction. It could be hypothesised that the patients with the least active disease will do better because of their better underlying natural history or conversely those who have the most active disease have the greatest potential for showing a response.

Increased disability, as measured by the HAQ score, was predictive for stopping treatment for adverse events, although not for inefficacy. The explanation for this finding is unclear. In other studies,^7^ higher levels of disability (as measured by the Steinbrocker functional class) were associated with a poorer response to treatment, which may be explained on the basis that chronic severe disease is less responsive to treatment, The association in the current study seen with adverse events may be explained by a high HAQ score being a marker for other psychosocial factors that may increase adverse events such as depression or altered illness beliefs. Previous studies have shown that the HAQ score, although accepted as one of the gold standard measures of disability in RA, is also associated with pain,^14^ depression and disease activity.^15^

In a meta-analysis, Anderson *et al*^7^ found that female patients had a poorer response to treatment. Although within the NOAR cohort, female patients were more likely to stop treatment for adverse events, the difference was not statistically significant. Other studies^9^ suggest that male patients are more likely to respond to MTX, although whether this relates to gender differences in MTX clearance or other factors remains unclear.

Interestingly, the strongest factor demonstrated was the association between being positive for the HLA DRB1 SE and stopping MTX for inefficacy. Small numbers limited the ability to be able to detect a dose effect, or the relative importance of different SE alleles. No association was observed between stopping MTX for adverse events and SE status. Other studies support a link between SE and MTX efficacy; O’Dell *et al*^16^ found that patients who were SE positive were less likely to respond to MTX monotherapy compared with combination treatment with MTX, sulfasalazine and hydroxychloroquine, whereas patients who were SE negative did equally well regardless of treatment allocation. Our results are in contrast to those of Criswell *et al*^17^ who found that patients who were SE positive were *more* likely to respond to MTX. Further studies are required to reconcile these apparently contradictory results.

The strength of this study is that we were able to recruit an unselected cohort of patients with IP and so the results are generalisable to other unselected inflammatory arthritis cohorts. The treatment of early IP is difficult because most of the evidence base relates to patients who satisfy the American College of Rheumatology (ACR) criteria. Yet it is increasingly recognised that treatment should be started as early as possible before the ACR criteria have been satisfied, with the PROMPT (Probable rheumatoid arthritis: Methotrexate versus Placebo Treatment) study showing benefit of early introduction of MTX in patients with undifferentiated IP in terms of slowing the progression to “ACR criteria” RA.^18^ Thus it is a strength of this study that it adds to the evidence base of the efficacy of treatment in patients with undifferentiated IP. There are, however, a number of limitations. First, the measure of disease response was not based on a formal protocol but reflected physician opinion. Hence the decision to stop treatment because of inefficacy was not standardised between patients. Further, as the NOAR follow-up was based on anniversary since entry, we do not have the detailed clinical data on disease activity and drug dose; for example, at the time of stopping treatment to obtain objective data to support the decision to stop treatment. This also meant that a number of patients were excluded from the analysis either because they had already started on MTX, or did not start within 3 months of their clinical assessment. Furthermore, there are additional, unmeasured predictors that may influence treatment response in this cohort, and hence improve the fit of the prediction models. We did not have radiographic data available on all patients and so these could not be included in the analysis. As this was a primary care based cohort of early disease, with short symptom duration, it was assumed that the prevalence of erosive disease at baseline would be low and, therefore, *x*-rays were not routinely taken on all patients at baseline.

Despite its limitations, this study highlights that clinical and laboratory factors per se, even in combination, are relatively poor at predicting treatment response to MTX at least as judged by physician decision to stop treatment. This suggests that, first, there is no simple decision rule that could guide which patients should and should not be commenced on MTX. Secondly, given that response to treatment is unlikely to be a truly random event, there must be a number of other biomarkers, for example, not measured in this study that may influence outcome of MTX treatment. There have been several studies suggesting that polymorphisms in some of the pathways involved in the metabolism of MTX may be useful in predicting drug outcome,^19^ with a recent pharmacogenetic model being used to predict drug outcome in a subset of patients.^20^ To date, the clinical utility of such a model has still to be fully established. Further attempts to refine our ability to predict treatment response may therefore need to incorporate both additional genetic biomarkers and psychosocial factors, such as compliance, patient education and illness beliefs, which may influence treatment response.

In summary, routinely gathered clinical and laboratory factors are poor at predicting outcome of treatment with MTX in patients with newly diagnosed inflammatory arthritis. Nevertheless, given the central importance of MTX in the management of RA further work is required to optimise our use of this treatment.
